# Neue Impfstoffe gegen Tuberkulose

**DOI:** 10.1007/s00103-019-03065-y

**Published:** 2019-11-29

**Authors:** Stefan H. E. Kaufmann

**Affiliations:** 1 Abteilung Immunologie, Max-Planck-Institut für Infektionsbiologie, Charitéplatz 1, 10117 Berlin, Deutschland; 2 Hagler Institute for Advanced Study, Texas A&M University, College Station, USA

**Keywords:** BCG, Impfung, Schutz vor Erkrankung, Schutz vor Infektion, Tuberkulose, BCG, Prevention of disease, Prevention of infection, Tuberculosis, Vaccination

## Abstract

Mit ca. 10 Mio. Erkrankungen und 1,5 Mio. Todesfällen im Jahr 2018 gehört die Tuberkulose (TB) weiterhin zu den bedrohlichsten Infektionskrankheiten weltweit. Dennoch erwartet die Weltgesundheitsorganisation (WHO), dass bis 2035 im Vergleich zu 2015 die Morbidität um 90 % und die Mortalität um 95 % gesenkt werden kann. Zwar stehen uns Diagnostika, Therapeutika und ein Impfstoff zur Verfügung, es besteht aber kein Zweifel, dass bessere Interventionsmaßnahmen benötigt werden, um dieses ehrgeizige Ziel zu erreichen. Der vorhandene Impfstoff Bacille Calmette-Guérin (BCG) schützt Kleinkinder teilweise gegen TB, ist aber weitgehend wirkungslos gegen Lungen-TB bei Jugendlichen und Erwachsenen. Die Möglichkeiten dieses Impfstoffs scheinen jedoch noch nicht voll ausgeschöpft zu sein. Zudem gibt es neue Impfstoffkandidaten, die sich derzeit in klinischer Überprüfung befinden.

Da ein Viertel der Menschheit mit *Mycobacterium tuberculosis* (*Mtb*) latent infiziert ist, müssen neue Impfstoffe nicht nur vor der Infektion (präexpositionell), sondern auch danach (postexpositionell) gegen die Erkrankung wirken. Als klinische Endpunkte werden Schutz vor Infektion, Schutz vor Erkrankung und Schutz vor Wiederauftreten (Rekurrenz) überprüft. Der Schutz gegen TB wird wesentlich von T‑Zell-Antworten getragen, weshalb in der Impfstoffentwicklung der Schwerpunkt hierauf gelegt wird. In der klinischen Überprüfung befinden sich Protein-Adjuvans-Impfstoffe, virale Vektoren, Tot- und Lebendimpfstoffe. Auch die Möglichkeit einer therapeutischen Impfung wird untersucht, um besonders bei multiresistenten TB-Fällen die Chemotherapie zu unterstützen. Es ist wahrscheinlich, dass ein einziger Impfstoff die verschiedenen Zielstellungen nicht erfüllen kann und unterschiedliche Impfstrategien benötigt werden.

## Abstract (en)

With about 10 million active disease cases and 1.5 million deaths in 2018, tuberculosis (TB) remains one of the most threatening infectious diseases. Yet, the World Health Organization (WHO) aims to reduce morbidity and mortality by 90 and 95%, respectively, between 2015 and 2035. Although diagnostics, therapeutics, and a vaccine are available, it is beyond doubt that better intervention measures are needed to accomplish this ambitious goal. The vaccine bacille Calmette-Guérin (BCG) partially protects infants against TB, but it is virtually ineffective against pulmonary TB in adolescents and adults. The efficacy of this vaccine, however, has not yet been fully exploited. In addition, new vaccine candidates are currently being assessed in clinical trials.

Because a quarter of all people are latently infected with *Mycobacterium tuberculosis* (*Mtb*), new vaccines must be applied not only prior to infection (pre-exposure vaccination) but also after infection (postexposure vaccination). Prevention of infection, prevention of disease, and prevention of recurrence are currently assessed as clinical endpoints. Because protection against TB is primarily mediated by T lymphocytes, TB vaccine development focuses on protective T cell responses. Protein adjuvant formulations, viral vectors, and killed and live bacterial vaccines are currently being assessed in clinical trials. Moreover, therapeutic vaccination is clinically tested, notably in adjunct to canonical drug therapy to multiresistant TB. It is likely that a single vaccine cannot accomplish the various indications and that different vaccination strategies are required.

## Einleitung

Weltweit bleibt der Erreger der Tuberkulose (TB), *Mycobacterium tuberculosis* (*Mtb*), die tödlichste Mikrobe [1]. Im Jahr 2018 starben 1,5 Mio. Menschen und 10 Mio. Menschen erkrankten neu. Man schätzt, dass weltweit ca. 1,7 Mrd. Menschen mit dem *Mtb*-Erreger infiziert sind, von denen im Laufe ihres Lebens etwa 10 % erkranken [2]. Die TB soll nach Vorstellungen der WHO bis 2035 deutlich zurückgedrängt werden: Im Vergleich zu 2015 wird angestrebt, die Mortalität um 95 % und die Morbidität um 90 % zu verringern [1, 3, 4]. Allgemein wird akzeptiert, dass dies nur gelingen kann, wenn die derzeit verfügbaren Möglichkeiten weltweit vollständig genutzt werden und gleichzeitig bessere Interventionsmaßnahmen, also bessere Medikamente, Diagnostika und Impfstoffe, entwickelt werden. Die Befürchtung, dass das Ziel der WHO nicht erreicht werden kann, besteht insbesondere auch deshalb, weil die Situation durch zwei Faktoren erschwert wird: Erstens sind Koinfektionen von HIV (humanes Immundefizienzvirus) und *Mtb *immer noch zu hoch; zweitens nehmen multiresistente („multi-drug-resistant“, MDR) und extensiv resistente („extensively drug-resistant“, XDR) TB-Fälle erschreckend zu [1]. Obwohl mit Bacille Calmette-Guérin (BCG) ein Impfstoff zur Verfügung steht, ist dessen Wirksamkeit ungenügend. Im Folgenden werden neue Impfstoffkandidaten gegen TB beschrieben, die sich derzeit in klinischer Überprüfung befinden.

## Immunpathologie der TB

**Figure 1:**
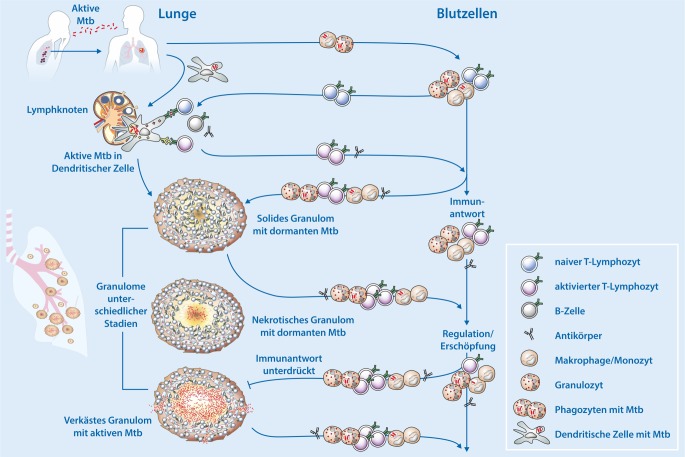

Am häufigsten wird die TB aerogen übertragen (Abb. 1). Patienten mit offener TB leiden unter schweren Hustenanfällen und Auswurf [2]. Dabei entstehen Aerosole mit Tröpfchenkernen von <5 µm Durchmesser, die *Mtb* enthalten. Personen in der Umgebung atmen Tröpfchen mit *Mtb*-Keimen ein, die auf diese Weise in die Alveolarräume der Lunge gelangen. Dort werden sie von professionellen Phagozyten, in erster Linie Makrophagen und Granulozyten im Alveolarraum, sowie dendritischen Zellen, die im Alveolarepithel eingebettet sind, aufgenommen [2, 5]. Die dendritischen Zellen transportieren die Erreger in die drainierenden Lymphknoten in der Lunge und die Alveolarmakrophagen verschleppen sie in das Lungengewebe. Dort vermehren sich die *Mtb*-Erreger und bilden einen Entzündungsherd. In den Lymphknoten stimulieren die dendritischen Zellen eine erworbene antigenspezifische Immunantwort, d. h., sie aktivieren T‑Lymphozyten und B‑Lymphozyten. T‑Lymphozyten rezirkulieren und gelangen so in die Lunge, wo sie am Ort der bakteriellen Ablagerung die Bildung eines soliden Granuloms induzieren [5–7]. B‑Lymphozyten verwandeln sich in Plasmazellen, die *Mtb-*spezifische Antikörper produzieren. Die T‑Zell-Antwort basiert in erster Linie auf spezifischen CD4-T-Lymphozyten vom T‑Helfer-1-Typ (TH-1). Wahrscheinlich werden auch spezifische TH-17-Zellen gebildet, die an der frühen Entzündung mitwirken. Zusätzlich entwickeln sich auch spezifische CD8-T-Zellen. Diese wie auch die TH-1-Lymphozyten produzieren die typischen Typ-I-Zytokine, d. h. Tumor-Nekrose-Faktor (TNF), Interferon‑γ (IFN-γ) und Interleukin‑2 (IL-2). Neben diesen Markerzytokinen werden bei der TB weitere Zytokine gebildet. Die CD8-T-Zellen sind auch zytolytisch, d. h., sie sind außerdem in der Lage, infizierte Makrophagen und auch *Mtb* direkt abzutöten.

Die entstandenen soliden Granulome mauern die *Mtb*-Keime ein und verhindern so die klinische Erkrankung (Abb. 1). In diesen Granulomen wandelt sich *Mtb* von einem metabolisch und replikativ aktiven Keim in einen dormanten Keim, der seine Lebensaktivitäten auf ein Minimum herunterfährt [6, 7]. Es entwickelt sich eine latente TB-Infektion (LTBI). Bei ca. 90 % der etwa 1,7 Mrd. LTBI-Fälle weltweit bleibt dieses Stadium lebenslang bestehen, d. h., die Personen erkranken nie [2, 8]. Bei ca. 10 % der Personen mit LTBI entwickelt sich im Laufe ihres Lebens eine aktive TB-Erkrankung. Bei knapp der Hälfte dieser Fälle bricht die TB innerhalb der ersten zwei Jahre aus. Bei den übrigen erst zu einem späteren Zeitpunkt.

Gerade den erst nach Jahren einsetzenden TB-Erkrankungen geht häufig eine Immunschwäche voraus. Diese kann (i) durch eine Koinfektion, (ii) durch eine Komorbidität und (iii) durch eine allgemeine Erschöpfung der Immunantwort angestoßen werden. Die wichtigste Koinfektion ist die Infektion mit HIV (humanes Immundefizienzvirus) und die TB ist die häufigste Todesursache von Patienten mit Aids (Acquired Immunodeficiency Syndrome; [1, 9]). Eine schwerwiegende Komorbidität für TB ist Diabetes mellitus [1, 9]. Die Schwächung bzw. Erschöpfung der Immunantwort wird durch verschiedene noch immer nicht ganz verstandene Mechanismen ausgelöst [10, 11]. Beteiligt sind myeloide Suppressorzellen („myeloid derived suppressor cells“) und regulatorische T‑Lymphozyten, welche inhibitorische Zytokine wie Interleukin-10 (IL-10) und TGF‑β (Transforming Growth Factor, transformierender Wachstumsfaktor) produzieren. Zusätzlich spielen auch direkte Zell-Zell-Interaktionen eine Rolle, an denen inhibitorische Oberflächenmoleküle, wie PD-1/PDL‑1 und CTLA-4/B7-Rezeptorpaare, beteiligt sind. Diese Interaktionen werden heute als Checkpoint Control bezeichnet und die Aufhebung ihrer inhibitorischen Wirkung wird bei der Krebstherapie erfolgreich genutzt [12].

Dem Krankheitsausbruch liegt auf Organebene die Umwandlung des soliden Granuloms in ein nekrotisches Granulom, das schließlich verkäst, zugrunde [6, 7]. In diesen Granulomen „wachen“ die *Mtb*-Keime wieder auf, werden stoffwechselaktiv und vermehren sich. Wenn das verkäsende Granulom in den Alveolarraum aufbricht, gelangen die Bakterien in die Umgebung – der TB-Patient ist ansteckend. Gelangen *Mtb*-Bakterien in den Blutstrom, können andere Organe infiziert werden, in denen sich dann ebenfalls Gewebeschäden entwickeln. Wir wissen heute, dass die drei Granulomformen bei der TB ein Kontinuum bilden und dass bei der Erkrankung alle Ausprägungen parallel vorkommen [6, 7]. Damit liegen auch die *Mtb*-Erreger bei der aktiven TB in unterschiedlichen Stadien vor. Dies ist ein Grund für die lange Behandlungsdauer von 6 Monaten. Während nämlich im soliden Granulom die *Mtb*-Keime dormant sind und daher sehr schlecht auf antibiotische Behandlung ansprechen, sind sie in den verkästen Granulomen aktiv und damit auch suszeptibel gegen TB-Medikamente [6, 7]. Weiterhin weisen neuere Untersuchungen darauf hin, dass der aktiven TB eine subklinische TB vorausgeht [13, 14]. Bei dieser Form der TB werden wahrscheinlich die Entzündungsreaktionen, welche von nekrotischen und verkästen Granulomen ausgehen, langsam stärker, wobei dieser Prozess im Laufe von 6 bis 12 Monaten in eine aktive TB mündet.

## BCG – der einzige lizenzierte TB-Impfstoff

Der bis heute einzige lizenzierte TB-Impfstoff, Bacille Calmette-Guérin (BCG), stammt vom Erreger der Rindertuberkulose, *Mycobacterium bovis*, ab [15]. Er wurde von den französischen Biomedizinern Albert Calmette und Camille Guérin zwischen 1906 und 1919 durch serielle Passage auf sterilen Kartoffelscheiben, die mit Ochsengalle getränkt waren, attenuiert [15]. Bereits 1908 konnten die Wissenschaftler feststellen, dass die 30. Passage in Tierexperimenten sicher war und Schutz vermittelte. 1919 wurde die 230. Passage in Kühen (dem natürlichen Wirt des ursprünglichen Erregers *M. bovis*) getestet. Auch dieses Experiment zeigte, dass der Impfstoff sicher und wirksam war. 1921 wurde daher erstmalig ein Neugeborenes, das in einer Familie mit einer TB-Patientin aufwuchs, geimpft. Das Kind blieb gesund. 1927 ergab eine retrospektive Analyse von über 20.000 geimpften Säuglingen, dass die Todesrate unter den geimpften Kleinkindern deutlich geringer war als in nichtgeimpften Neugeborenen (obwohl dies keine klinische Studie nach unseren heutigen Maßstäben darstellt; [15]). In den darauffolgenden Jahrzehnten wurde die BCG-Impfung weltweit immer häufiger eingesetzt. Generell wird der BCG-Impfstoff intradermal verabreicht. Dieser Applikationsweg wird auch für die neuen TB-Impfstoffe bevorzugt genutzt, obwohl auch alternative Wege, insbesondere per Aerosol, diskutiert werden. Heute ist der BCG-Impfstoff Teil des EPI (Expanded Program for Immunization) für Kleinkinder mit einer Impfdecke von >80 % in Ländern, in denen die BCG-Impfung empfohlen wird. Circa 100 Mio. Kinder werden jährlich mit BCG geimpft und mit mehr als 4 Mrd. Dosen ist die BCG-Impfung die am häufigsten durchgeführte Impfung weltweit. Metaanalysen zur Impfeffektivität zeigen, dass der BCG-Impfstoff in Kleinkindern einen moderaten Schutz gegen TB bietet; gegen die häufigste Form der TB, die Lungenerkrankung, schützt der Impfstoff jedoch insbesondere Jugendliche und Erwachsene nicht verlässlich [16–18]. Daher wird allgemein akzeptiert, dass bessere TB-Impfstoffe dringend benötigt werden, um die weltweite Bedrohung der TB einzudämmen und sie sogar zu eliminieren.

## Schutz von Infektion durch Zweitimpfung mit BCG

Im Jahr 2018 wurde eine klinische Studie zur Prävention der *Mtb*-Infektion mit BCG abgeschlossen [19, 20]. Getestet wurde neben BCG ein neuer Impfstoff (der adjuvantierte Proteinimpfstoff H4:IC31). Die Studienteilnehmer waren weder mit *Mtb* noch mit HIV infiziert. Sie waren in ihrer Kindheit bereits mit BCG geimpft worden; erhielten also eine Zweitimmunisierung mit BCG. Als klinischer Endpunkt diente die Konversion im immunologischen Gamma-Interferon-Test (IGRA) als Zeichen für eine Infektion mit *Mtb*. Der IGRA ermittelt die *Mtb*-spezifische IFN-γ-Produktion durch T‑Lymphozyten nach Restimulierung mit Mtb-spezifischen Antigenen, die BCG fehlen. Diese Studie ergab eine ca. 45 % niedrigere IGRA-Konversion nach Zweitimmunisierung mit BCG gegenüber Kontrollen. Keine Wirkung wurde für den H4:IC31-Impfstoff festgestellt, dessen Weiterentwicklung daher eingestellt werden soll [19, 20].

Grundsätzlich bietet diese klinische Studie aber Hinweise auf einen Schutz gegen Infektion durch eine Zweitimmunisierung mit BCG. Der Befund, dass anfangs kein Schutz gegen Infektion gegenüber den Kontrollen festzustellen war, sondern erst nach einem längeren Zeitraum, deutet darauf hin, dass die Infektion anfangs nicht verhindert wurde. Vielmehr liegt nahe, dass die Zweitimmunisierung T‑Zellen aktivierte, die den Erreger während der frühen Infektion eliminieren, also eine transiente Infektion zulassen, aber die stabile Infektion verhindern. Theoretisch ist auch möglich, dass die Zweitimmunisierung das angeborene Immunsystem so trainierte, dass Makrophagen T‑zell-unabhängig die *Mtb*-Erreger eliminierten. Ein Trainingseffekt aufgrund epigenetischer Veränderungen durch BCG wurde in Experimentalmodellen beschrieben und als Erklärung für die Verringerung der generellen Morbidität und Mortalität durch BCG in Ländern mit niedrigstem Einkommen herangezogen [21].

## Übersicht über das Portfolio an TB-Impfstoffkandidaten

Die oben beschriebenen Mechanismen bilden die Blaupause für die Entwicklung neuer Impfstoffkandidaten [19, 22, 23]. In erster Linie wird angestrebt, eine stärkere T‑Zell-Immunität durch die Impfung zu stimulieren, die von einer Antikörperantwort unterstützt wird. Der Schwerpunkt liegt dabei auf der Stimulation von CD4-TH-1-Zellen, begleitet von einer zusätzlichen kontrollierten Bildung von CD4-TH-17-Zellen und CD8-T-Zellen [5, 22]. Daneben soll auch die spezifische Antikörperproduktion angeregt werden [24]. Eine „verbesserte“ Immunantwort kann durch geeignete Adjuvanzien, virale Träger, abgetötete Mykobakterien oder Lebendimpfstoffe erreicht werden (Tab. 1).

**Table 1:** 

PRÄVENTIVIMPFSTOFFE	Typ	Identifikationsnummer in Datenbank *ClinicalTrials.*gov (Phase einer repräsentativen Studie)
*Adjuvantierte Protein-Impfstoffe*		
M72: AS01E	Fusionsprotein/Adjuvans	NCT 01755598 (IIb, abgeschlossen)
H56: IC31	Fusionsprotein/Adjuvans	NCT 03512249 (II)
ID93: GLA-SE	Fusionsprotein/Adjuvans	NCT 03806686 (II)
*Vektorbasierte Impfstoffe*		
TB-FLU-04L	Replikationsdefiziente Influenzaviren (H1N1)	NCT 02501421 (I, abgeschlossen)
MVA85A (intradermal, Aerosol)	Modifiziertes Vaccinia-Ankara-Virus	NCT 01954563 (I, abgeschlossen)
Ad5Ag85A	Replikationsdefizientes Adenovirus Vektor	NCT 02337270 (I)
ChAdOx1.85A + MVA85A	Schimpansenadenovirus + modifiziertes Vaccinia-Ankara-Virus	NCT 03681860 (II)
*Totimpfstoffe*		
Vaccae	Abgetötete *M.-vaccae-*Keime	NCT 01979900 (III)
Dar-901	Abgetötete *M.-obuense-*Keime	NCT 02712424 (II)
*M. indicus pranii *(MIP)	Abgetötete *M.-indicus-pranii-*Keime	NCT 00265226 (III)
*Lebendimpfstoffe*		
VPM1002	Rekombinanter BCG-Impfstoff (rBCG)	NCT 03152903 (III)
MTBVAC	Genetisch attenuierte *M. tuberculosis*	NCT 02933281 (II)

^a^Nichtweiterentwickelte Impfstoffe werden nicht aufgeführt.

In der klinischen Überprüfung befinden sich:

- **adjuvantierte Fusionsproteine**, die aus zwei oder mehreren *Mtb*-Antigenen bestehen und mit einem potenten Adjuvans zur T‑Zell-Aktivierung formuliert werden. Da *Mtb* in der dormanten Phase andere Antigene exprimiert als in der aktiven Phase, versucht man in die Impfstoffkandidaten Antigene der unterschiedlichen Stadien zu integrieren [22]. Man spricht dann von einem „Multistadienimpfstoff“ (Multi Stage Vaccine).
  Hierzu gehören M72:AS01, H56:IC31 und ID93:GLA-SE (Tab. 1, Tab. 2 und Tab. 3; [25–27]).
- **virale Vektoren**, die ein Antigen von *Mtb* exprimieren. Als Vektoren dienen replikationsunfähige Influenzaviren, modifizierte Vaccinia-Ankara-Viren (MVA) und humane oder Schimpansenadenoviren. Hierzu zählen TB-FLU-04L, MVA85A, Ad5Ag85A und ChAdOx.185A (Tab. 1 und Tab. 2; [28–32]).
- **Totimpfstoffe**, die aus atypischen Mykobakterien oder teilgereinigten *Mtb*-Bestandteilen bestehen. Hierzu zählen *Mycobacterium vaccae *(Vaccae), *Mycobacterium obuense* (Dar-901) und *Mycobacterium indicus pranii* (MIP; Tab. 1) sowie der Impfstoff RUTI, der aus teilgereinigten *Mtb*-Bestandteilen aufgebaut ist (Tab. 4; [33–36]).
- **Lebendimpfstoffe** besitzen weitgehend alle Antigene des *Mtb*-Erregers. Untersucht werden verbesserte BCG-Impfstoffe, die durch genetische Modifikation eine verstärkte Immunantwort auslösen oder Deletionsmutanten von *Mtb*, die die Immunantwort gegen TB imitieren, ohne Schäden hervorzurufen. Hierzu zählen der rekombinante BCG-Impfstamm VPM1002 und der attenuierte *Mtb*-Impfstamm MTBVAC (Tab. 1; [37–41]).

**Table 2:** 

Impfstoff	Antigene	Kurzbeschreibung
M72	Rv1106 Rv0125	PPE-Familie Peptidase
H56	ESAT‑6 Ag85b Rv2660c	Prominentes Antigen (RD1) Mycolyltransferase Dormanzantigen
ID93	Rv2608 Rv3619 Rv3620 Rv1813	PPE-Familie Virulenzfaktor (RD1) Virulenzfaktor (RD1) Dormanzantigen
Ad5Ag85A	Antigen 85A	Mycolyltransferase
MVA85A	Antigen 85A	Mycolyltransferase
ChAdOx1.85A	Antigen 85A	Mycolyltransferase
TB-FLU-04L	Antigen 85A ESAT‑6	Mycolyltransferase Prominentes Antigen (RD1)

**Table 3:** 

Impfstoff	Adjuvans	Zusammensetzung
H56	IC31	Kationische Peptide + TLR9-Agonist
ID93	GLA-SE	Squalenöl in Wasseremulsion + TLR4-Agonist
M72	AS01E	Liposomen mit Monophosphoryl Lipid A + Saponin (QS21)

**Table 4:** 

Impfstoff	Strategie	Typ
*M. indicus pranii (M w)*	Therapeutisch (plus Chemotherapie)	Abgetötete *M.-indicus-pranii-*Keime
Vaccae	Therapeutisch (plus Chemotherapie)	Abgetötete *M.-vaccae-*Keime
RUTI	Therapeutisch (plus Chemotherapie)	Abgetötete und teilgereinigte *Mtb*-Keime
H56: IC31	Therapeutisch (plus Chemotherapie)	Fusionsprotein/Adjuvans

Die Diagnose einer Mtb-Infektion mithilfe von IGRA stellt einen wesentlichen Teil der TB-Kontrolle dar. Impfstoffe, welche Antigene der verwendeten IGRA enthalten, würden daher ein falsch-positives Resultat hervorrufen. Dies stände möglicherweise einer Einführung solcher Impfstoffe im Wege. Es sei denn, es werden neue IGRA entwickelt, die auf diese Antigene verzichten. Dies gilt für den Lebendimpfstoff MTBVAC, den adjuvantierten Proteinimpfstoff H56:IC31 und den viralen Vektor TB-FLU-04L.

**Figure 2:**
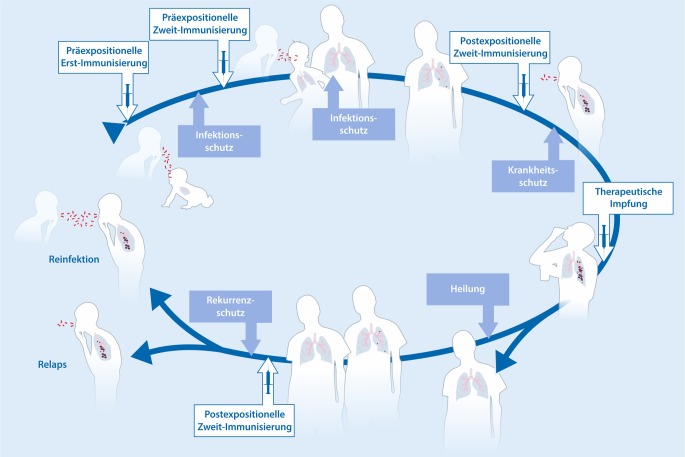

Präventivimpfstoffe werden entweder präexpositionell, also vor der *Mtb*-Infektion oder postexpositionell, also nach der Infektion verabreicht (Abb. 2). Die Präexpositionsimpfung ist insbesondere für Kleinkinder vorgesehen, während die Postexpositionsimpfung in erster Linie für Heranwachsende und Erwachsene geeignet ist, da diese in endemischen Gebieten häufig bereits infiziert sind und damit als LTBI-Fälle einzustufen sind. Ein Impfstoff zur Erstimmunisierung von Neugeborenen soll den BCG-Impfstoff ersetzen. Die meisten Impfstoffe sind zur Zweitimmunisierung vorgesehen und sollen die Erstimmunisierung mit BCG verbessern. Impfstoffe zur Erstimmunisierung werden auch für Zweitimmunisierungen erwogen. Längerfristig wird die Erstimmunisierung mit einem neuen Impfstoffkandidaten, gefolgt von der Zweitimmunisierung mit einem heterologen Kandidaten, angedacht.

Die Impfung von Neugeborenen hat zum Ziel, die Infektion und/oder die aktive Erkrankung zu verhindern (Abb. 2). Dies ist auch das Ziel der Zweitimmunisierung von Heranwachsenden und Erwachsenen, die noch nicht mit *Mtb* infiziert sind. Da die meisten Personen in endemischen Gebieten bereits infiziert sind, kann bei diesen die Infektion nicht verhindert werden. Der Schwerpunkt liegt daher auf der Prävention der Krankheit.

Schließlich wird die Prävention einer Rekurrenz (Schutz vor Wiedererkrankung) in klinischen Studien überprüft. In endemischen Gebieten erkranken ca. 10 % aller Tuberkulosepatienten trotz apparenter Heilung nach medikamentöser Behandlung innerhalb von 12 Monaten erneut [43]. Es wird geschätzt, dass dies bei >60 % auf Relaps (Erkrankungsrückfall) und bei <40 % auf erneute Infektion zurückzuführen ist. Neuere Daten weisen auf eine schützende Nische für *Mtb* im Knochenmark hin, die als Ausgangspunkt eines Erkrankungsrückfalls infrage kommt [44, 45]. Die Reinfektion erfolgt mit einem neuen *Mtb*-Stamm. Zwar ist Schutz vor einer Wiedererkrankung nicht das Hauptanliegen einer TB-Impfung, die hohe Erkrankungsrate innerhalb eines überschaubaren Zeitraums ermöglicht allerdings eine rasche Impfstoffprüfung an relativ geringen Probandenzahlen. Die häufige Reinfektion nach erfolgreicher TB-Behandlung kann als Anzeichen gedeutet werden, dass zumindest bei den betroffenen Individuen die *Mtb*-Infektion keinen zufriedenstellenden Schutz hervorruft. Es wird deshalb allgemein die Meinung vertreten, dass ein erfolgreicher neuer Impfstoff eine quantitativ stärkere oder qualitativ andere protektive Immunantwort hervorrufen muss.

Obwohl der Schwerpunkt der TB-Impfstoffentwicklung weiterhin auf der Prävention liegt, werden auch therapeutische Impfstoffe entwickelt, die eine Chemotherapie unterstützen sollen. Dies ist besonders für die Behandlung der multiresistenten TB wichtig [42]. In erster Linie sind dies Totimpfstoffe, obwohl auch andere Impfstoffe, die primär zur Prävention entwickelt werden, dafür in Betracht gezogen werden (Tab. 4).

## Klinische Studien mit adjuvantierten Proteinimpfstoffen

Der am weitesten fortgeschrittene adjuvantierte Proteinimpfstoff ist M72:AS01 [27]. Er wurde kürzlich auf Schutz gegen Erkrankung in gesunden HIV-negativen Erwachsenen, die zu Beginn der Studie bereits eine LTBI hatten, untersucht. Als klinischer Endpunkt diente der Nachweis von *Mtb* im Sputum als Indikator einer Lungen-TB. Die Auswertung nach zweijährigem Studienverlauf ergab einen ca. 50 %igen Schutz durch M72:AS01 gegenüber Kontrollen, also nichtgeimpften Studienteilnehmern. Eine Vergleichskontrolle mit BCG fehlte in dieser Studie.

Die adjuvantierten Proteinimpfstoffe ID93:GLA-SE und H56:IC31 werden auf Prävention von Infektion und Erkrankung sowie auf Prävention der Rekurrenz getestet [22]. Der Impfstoff ID93 besteht aus einem tetravalenten Fusionsprotein, dass zwei Virulenzfaktoren (Rv3619, Rv3620) sowie ein Mitglied der Prolin-Prolin-Glutamin-(PPE-)Proteinfamilie (Rv2608) und ein Dormanzantigen (Rv1813) umfasst (Tab. 2). Das eingesetzte Adjuvans stellt eine Öl-in-Wasser-Emulsion dar, in die ein Agonist für den Toll-like-Rezeptor (TLR) 4 eingebettet ist (Tab. 3). Der Impfstoff H56:IC31 umfasst ein trivalentes Fusionsprotein und ein Adjuvans aus kationischen Peptiden und einem TLR-9-Agonisten. Beide Impfstoffe haben ihre Sicherheit und Immunogenität in Erwachsenen in klinischen Phase-I-Studien belegt und sollen auf Schutz vor Infektion und/oder Erkrankung nach Zweitimmunisierung geprüft werden.

## Klinische Studien mit viralen Vektoren

Impfstoffe mit viralen Vektoren, die ein oder mehrere Antigene von *Mtb* exprimieren, sind humane oder Schimpansenadenoviren, das MVA oder ein replikationsdefizientes H1N1-Grippevirus (Tab. 1; [28–32]). Der MVA-Impfstoffträger exprimiert das Antigen (Ag) 85A. Die Ag85-Familie umfasst drei Mitglieder, die Ag85A, Ag85B und Ag85C genannt werden (Tab. 2). Der erste neue TB-Impfstoff, der auf Schutz vor Erkrankung getestet wurde, war der virale Impfstoffträger MVA, der ein einzelnes Antigen exprimierte (Ag85A). Dieser Impfstoff enttäuschte aber, da er nachweislich keinen Schutz hervorrief [46].

Nachdem MVA Ag85A keinen Schutz zeigte, werden nun andere Darreichungsformen für diesen Kandidaten geprüft: (i) eine Aerosolimpfung; (ii) eine intradermale Erstimmunisierung gefolgt von einer Aerosolimpfung sowie eine Zweitimmunisierung, die auf eine Schimpansenadenoviruserstimmunisierung folgt (ChAdOx1.85A → MVA85A; [28, 29]). Ein humanes replikationsdefizientes Adenovirus 5, das ebenfalls Ag85A exprimiert, befindet sich ebenfalls in klinischer Testung (Ad5Ag85A; [31]). Wenig ist über den influenzavirusbasierten Impfstoff bekannt. Hierbei handelt es sich um ein replikationsdefizientes H1N1-Influenzavirus, das neben Ag85A auch den Virulenzfaktor ESAT‑6 exprimiert [30]. ESAT‑6 ist ein prominentes Virulenzgen von *Mtb*, das in BCG nicht vorkommt (Tab. 2).

## Klinische Studien mit Impfstoffen aus abgetöteten Mykobakterien

Drei Impfstoffkandidaten aus abgetöteten atypischen Mykobakterien befinden sich in der klinischen Testung (Tab. 1). Die Informationen dazu sind jedoch spärlich oder widersprüchlich. Dies ist erstens *Mycobacterium vaccae* (kurz Vaccae), das bereits 2017 eine Phase-III-Studie in China abgeschlossen haben soll [36]. Allerdings sind bislang keine Daten öffentlich verfügbar.

Ein weiterer Kandidat ist *Mycobacterium indicus pranii* (MIP; auch unter dem Kürzel Mw geführt). In einer Post-hoc-Analyse von Daten aus einer Leprapräventionsstudie wurde das Auftreten einer TB-Erkrankung in der Studienpopulation ausgewertet [34, 47, 48]. Die Analyse ergab einen gewissen Schutz gegen TB-Erkrankung. Der MIP-Impfstoff befindet sich derzeit zusammen mit VPM1002 in einer vergleichenden Phase-III-Studie (s. unten).

Der dritte Impfstoff ist *Mycobacterium obuense* (ursprünglich SRL-172 genannt), der in der sogenannten Dar-Dar-Studie auf Schutz gegen TB in HIV-positiven Erwachsenen einen mäßigen Schutz gegen TB-Erkrankung erzielte [49]. Ursprünglich war dieser Impfstamm als *Mycobacterium vaccae* typisiert; er wurde aus verschiedenen Gründen nicht weiterentwickelt. Schließlich wurden die Anzuchtbedingungen für den Impfstoff geändert. Der nun als Dar-901 bezeichnete Impfstoff befindet sich derzeit in klinischen Studien [35].

## Klinische Studien mit Lebendimpfstoffen

Lebendimpfstoffe in der klinischen Untersuchung sind derzeit der BCG-basierte rekombinante Impfstoff VPM1002 und der gendeletierte *Mtb*-basierte Impfstoff MTBVAC (Tab. 1).

Beim Impfstoff VPM1002 wurde das Gen, das die Urease C codiert, durch das heterologe Gen für Listeriolysin aus *Listeria monocytogenes* ersetzt [50]. Auf diese Weise wurde in präklinischen Studien ein äußerst effektiver und sicherer Impfstoff gewonnen. In zwei Phase-I-Studien, eine in Deutschland und eine in Südafrika, erwies sich der Impfstoff für junge Erwachsene als sicher und immunogen [38]. Auch in Kleinkindern konnte er seine Sicherheit und Immunogenität in einer Phase-IIa-Studie belegen [39]. Eine Phase-II-Studie mit HIV-exponierten und nichtexponierten Kleinkindern ist abgeschlossen, aber noch nicht entblindet. VPM1002 befindet sich derzeit in einer Phase-III-Studie auf Schutz gegen Rekurrenz.

Im Sommer 2019 hat der Indian Council of Medical Research eine multizentrische Phase-III-Studie begonnen, in der der Schutz von Haushaltskontakten von TB-Patienten durch eine Impfung mit VPM1002 oder MIP parallel überprüft wird. Im Jahr 2019 soll weiterhin eine Phase-III-Studie mit VPM1002 auf Schutz vor Infektion in HIV-exponierten und nichtexponierten Kleinkindern in Subsahara-Afrika beginnen. Weil bei Neugeborenen mit Immunsuppression einschließlich HIV-Infektion eine BCG-Impfung von der WHO als kontraindiziert eingestuft wird, wird die BCG-Impfung von HIV-exponierten Kindern auch nicht befürwortet [51]. Da der Impfstoff VPM1002 in präklinischen Studien [50] deutlich sicherer als BCG ist, soll in dieser Studie auch die Sicherheit des neuen Lebendimpfstoffs bei HIV-Exposition bestimmt werden.

Beim Impfstoff MTBVAC wurden in *Mtb* zwei Gene, die sich als zentrale Schaltstellen für die Expression zahlreicher Virulenzgene darstellen, deletiert [37, 41]. Dies sind *phoP*, ein Transkriptionsfaktor für Virulenzfaktoren von *Mtb* und fadD26, das an der Synthese eines Lipids von *Mtb* mitwirkt. Dieser Impfstoff erwies sich in einer Phase-I-Studie in Erwachsenen als sicher [41] und wird in einer Phase-II-Studie in Kleinkindern in Subsahara-Afrika auf Sicherheit und Immunogenität getestet werden.

## Ausblick

Nach langer Zeit des Stillstands hat die Forschung und Entwicklung von Impfstoffen gegen Tuberkulose Fahrt aufgenommen. Auf der Basis neuer Erkenntnisse in Genetik, Mikrobiologie und Immunologie wurden neue Impfkonzepte entwickelt, experimentell überprüft und in präklinischen und klinischen Studien getestet. Unterstützt wird diese Vorgehensweise durch generell akzeptierte Richtlinien, die über die unterschiedlichen Entwicklungsphasen festlegen, welche Voraussetzungen erfüllt sein müssen, um den Impfstoff in der folgenden Stufe weiterzuentwickeln [52].

Auf diese Weise konnten im letzten Jahrzehnt etwa ein Dutzend Impfstoffe in klinische Studien überführt werden, von denen einige bereits auf Sicherheit und Wirksamkeit getestet werden oder diese Meilensteine bereits überwunden haben. Es ist unwahrscheinlich, dass ein einziger Impfstoff sämtliche Herausforderungen meistern wird. Eher ist wahrscheinlich, dass unterschiedliche Impfstoffe zur Präexpositionsimpfung von Neugeborenen auf Schutz vor Infektion und Erkrankung und zur Postexpositionsimpfung von Erwachsenen mit LTBI auf Schutz vor Erkrankung benötigt werden. Neben diesen beiden Gruppen werden auch die Impfung von Erwachsenen ohne LTBI auf Schutz vor Infektion und Erkrankung sowie die Impfung auf Schutz vor Rekurrenz bei medikamentös geheilten TB-Patienten weiterverfolgt. Selbst wenn ein Impfstoff für eine bestimmte Anwendung in den nächsten Jahren zugelassen werden sollte, bedeutet dies nicht den Abschluss von Forschung und Entwicklung neuer Impfstoffe gegen TB. Abgeschlossene Impfstudien können zusätzlich Hinweise darauf ergeben, warum bestimmte Personenkreise geschützt sind und andere nicht. Darauf aufbauend können gezielt Impfstoffe der nächsten Generation entwickelt werden [53].

## References

[1] World Health Organization (2019). WHO global tuberculosis report 2019.

[2] Pai M, Behr MA, Dowdy D (2016). Tuberculosis. Nat Rev Dis Primers.

[3] World Health Organization. (2015) The end TB strategy. http://www.who.int/tb/strateg/end-tb/en/. Zugegriffen: 4. Nov. 2019

[4] Stop TB Partnership (2016) The global plan to end TB: 2016–2020, the paradigm shift. http://www.stoptb.org/assets/documents/global/plan/GlobalPlanToEndTB_TheParadigmShift_2016-2020_StopTBPartnership.pdf. Zugegriffen: 4. Nov. 2019

[5] Ottenhoff TH, Kaufmann SH (2012). Vaccines against tuberculosis: Where are we and where do we need to go?. PLoS Pathog.

[6] Barry CE, Boshoff HI, Dartois V (2009). The spectrum of latent tuberculosis: rethinking the biology and intervention strategies. Nat Rev Microbiol.

[7] Gengenbacher M, Kaufmann SHE (2012). Mycobacterium tuberculosis: success through dormancy. FEMS Microbiol Rev.

[8] Houben RM, Dodd PJ (2016). The global burden of latent tuberculosis infection: a re-estimation using mathematical modelling. PLoS Med.

[9] Ronacher K, Joosten SA, van Crevel R (2015). Acquired immunodeficiencies and tuberculosis: focus on HIV/AIDS and diabetes mellitus. Immunol Rev.

[10] Dorhoi A, Du Plessis N (2017). Monocytic myeloid-derived suppressor cells in chronic infections. Front Immunol.

[11] Urdahl KB (2014). Understanding and overcoming the barriers to T cell-mediated immunity against tuberculosis. Semin Immunol.

[12] Wei SC, Duffy CR, Allison JP (2018). Fundamental mechanisms of immune checkpoint blockade therapy. Cancer Discov.

[13] Suliman S, Thompson E, Sutherland J (2018). Four-gene pan-African blood signature predicts progression to tuberculosis. Am J Respir Crit Care Med.

[14] Zak DE, Penn-Nicholson A, Scriba TJ (2016). A blood RNA signature for tuberculosis disease risk: a prospective cohort study. Lancet.

[15] Calmette A, Guérin C, Boquet A, Négre L (1927). La vaccination préventive contre la tuberculose par le “BCG”.

[16] Colditz GA, Berkey CS, Mosteller F (1995). The efficacy of bacillus Calmette-Guerin vaccination of newborns and infants in the prevention of tuberculosis: meta-analyses of the published literature. Pediatrics.

[17] Colditz GA, Brewer TF, Berkey CS (1994). Efficacy of BCG vaccine in the prevention of tuberculosis. Meta-analysis of the published literature. JAMA.

[18] Mangtani P, Abubakar I, Ariti C (2014). Protection by BCG vaccine against tuberculosis: a systematic review of randomized controlled trials. Clin Infect Dis.

[19] Ginsberg AM (2019). Designing tuberculosis vaccine efficacy trials—lessons from recent studies. Expert Rev Vaccines.

[20] Nemes E, Geldenhuys H, Rozot V (2018). Prevention of M. tuberculosis infection with H4:IC31 vaccine or BCG revaccination. N Engl J Med.

[21] de Bree LCJ, Koeken V, Joosten LAB (2018). Non-specific effects of vaccines: current evidence and potential implications. Semin Immunol.

[22] Andersen P, Scriba TJ (2019). Moving tuberculosis vaccines from theory to practice. Nat Rev Immunol.

[23] Kaufmann SH, Weiner J, von Reyn CF (2017). Novel approaches to tuberculosis vaccine development. Int J Infect Dis.

[24] Li H, Javid B (2018). Antibodies and tuberculosis: finally coming of age?. Nat Rev Immunol.

[25] Penn-Nicholson A, Tameris M, Smit E (2018). Safety and immunogenicity of the novel tuberculosis vaccine ID93 + GLA-SE in BCG-vaccinated healthy adults in South Africa: a randomised, double-blind, placebo-controlled phase 1 trial. Lancet Respir Med.

[26] Suliman S, Luabeya AKK, Geldenhuys H (2019). Dose optimization of H56:IC31 vaccine for tuberculosis-endemic populations. A double-blind, placebo-controlled, dose-selection trial. Am J Respir Crit Care Med.

[27] Van Der Meeren O, Hatherill M, Nduba V (2018). Phase 2b controlled trial of M72/AS01E vaccine to prevent tuberculosis. N Engl J Med.

[28] Manjaly Thomas Z-R, Satti I, Marshall JL (2019). Alternate aerosol and systemic immunisation with a recombinant viral vector for tuberculosis, MVA85A: a phase I randomised controlled trial. PLoS Med.

[29] Satti I, Meyer J, Harris SA (2014). Safety and immunogenicity of a candidate tuberculosis vaccine MVA85A delivered by aerosol in BCG-vaccinated healthy adults: a phase 1, double-blind, randomised controlled trial. Lancet Infect Dis.

[30] Sergeeva MV, Pulkina AA, Vasiliev KA (2017). Safety and immunogenicity of cold-adapted recombinant influenza vector expressing ESAT-6 and Ag85A antigens of M. tuberculosis. Vopr Virusol.

[31] Smaill F, Jeyanathan M, Smieja M (2013). A human type 5 adenovirus-based tuberculosis vaccine induces robust T cell responses in humans despite preexisting anti-adenovirus immunity. Sci Transl Med.

[32] Stylianou E, Griffiths KL, Poyntz HC (2015). Improvement of BCG protective efficacy with a novel chimpanzee adenovirus and a modified vaccinia Ankara virus both expressing Ag85A. Vaccine.

[33] Nell AS, D’Lom E, Bouic P (2014). Safety, tolerability, and immunogenicity of the novel antituberculous vaccine RUTI: randomized, placebo-controlled phase II clinical trial in patients with latent tuberculosis infection. PLoS ONE.

[34] Sharma SK, Katoch K, Sarin R (2017). Efficacy and safety of Mycobacterium indicus pranii as an adjunct therapy in category II pulmonary tuberculosis in a randomized trial. Sci Rep.

[35] von Reyn CF, Lahey T, Arbeit RD (2017). Safety and immunogenicity of an inactivated whole cell tuberculosis vaccine booster in adults primed with BCG: a randomized, controlled trial of DAR-901. PLoS ONE.

[36] Weng H, Huang J-Y, Meng X-Y, Li S, Zhang G-Q (2016). Adjunctive therapy of Mycobacterium vaccae vaccine in the treatment of multidrug-resistant tuberculosis: a systematic review and meta-analysis. Biomed Rep.

[37] Aguilo N, Gonzalo-Asensio J, Alvarez-Arguedas S (2017). Reactogenicity to major tuberculosis antigens absent in BCG is linked to improved protection against Mycobacterium tuberculosis. Nat Commun.

[38] Grode L, Ganoza CA, Brohm C (2013). Safety and immunogenicity of the recombinant BCG vaccine VPM1002 in a phase 1 open-label randomized clinical trial. Vaccine.

[39] Loxton AG, Knaul JK, Grode L (2017). Safety and immunogenicity of the recombinant Mycobacterium bovis BCG vaccine VPM1002 in HIV-unexposed newborn infants in South Africa. Clin Vaccine Immunol.

[40] Nieuwenhuizen NE, Kulkarni PS, Shaligram U (2017). The recombinant bacille Calmette-Guerin vaccine VPM1002: ready for clinical efficacy testing. Front Immunol.

[41] Spertini F, Audran R, Chakour R (2015). Safety of human immunisation with a live-attenuated Mycobacterium tuberculosis vaccine: a randomised, double-blind, controlled phase I trial. Lancet Respir Med.

[42] Cardona PJ (2006). RUTI: a new chance to shorten the treatment of latent tuberculosis infection. Tuberculosis.

[43] Rosser A, Marx FM, Pareek M (2018). Recurrent tuberculosis in the pre-elimination era. Int J Tuberc Lung Dis.

[44] Reece ST, Vogelzang A, Tornack J (2018). Mycobacterium tuberculosis-infected hematopoietic stem and progenitor cells unable to express inducible nitric oxide synthase propagate tuberculosis in mice. J Infect Dis.

[45] Tornack J, Reece ST, Bauer WM (2017). Human and mouse hematopoietic stem cells are a depot for dormant Mycobacterium tuberculosis. PLoS ONE.

[46] Tameris MD, Hatherill M, Landry BS (2013). Safety and efficacy of MVA85A, a new tuberculosis vaccine, in infants previously vaccinated with BCG: a randomised, placebo-controlled phase 2b trial. Lancet.

[47] Katoch K, Singh P, Adhikari T (2008). Potential of Mw as a prophylactic vaccine against pulmonary tuberculosis. Vaccine.

[48] Kamal R, Pathak V, Kumari A (2017). Addition of Mycobacterium indicus pranii vaccine as an immunotherapeutic to standard chemotherapy in borderline leprosy: a double-blind study to assess clinical improvement (preliminary report). Br J Dermatol.

[49] Lahey T, Arbeit RD, Bakari M (2010). Immunogenicity of a protective whole cell mycobacterial vaccine in HIV-infected adults: a phase III study in Tanzania. Vaccine.

[50] Grode L, Seiler P, Baumann S (2005). Increased vaccine efficacy against tuberculosis of recombinant Mycobacterium bovis bacille Calmette-Guérin mutants that secrete listeriolysin. J Clin Invest.

[51] World Health Organization (2007). Revised BCG vaccination guidelines for infants at risk for HIV infection. Wkly Epidemiol Rec.

[52] Tuberculosis Vaccine Initiative (2019) Homepage https://www.tbvi.eu/. Zugegriffen: 4. Nov. 2019

[53] Kaufmann SH, Evans TG, Hanekom WA (2015). Tuberculosis vaccines: time for a global strategy. Sci Transl Med.

